# Optimal environmental testing frequency for outbreak surveillance

**DOI:** 10.1016/j.epidem.2024.100750

**Published:** 2024-02-15

**Authors:** Jason W. Olejarz, Kirstin I. Oliveira Roster, Stephen M. Kissler, Marc Lipsitch, Yonatan H. Grad

**Affiliations:** a Department of Immunology and Infectious Diseases, Harvard T. H. Chan School of Public Health, Boston, MA 02115, USA; b Center for Communicable Disease Dynamics, Harvard T. H. Chan School of Public Health, Boston, MA 02115, USA; c Department of Computer Science, University of Colorado Boulder, Boulder, CO 80309, USA; d Department of Epidemiology, Harvard T. H. Chan School of Public Health, Boston, MA 02115, USA

**Keywords:** Environmental surveillance, Early pathogen detection, Wastewater sampling, Vector trapping, Mathematical modeling

## Abstract

Public health surveillance for pathogens presents an optimization problem: we require enough sampling to identify intervention-triggering shifts in pathogen epidemiology, such as new introductions or sudden increases in prevalence, but not so much that costs due to surveillance itself outweigh those from pathogen-associated illness. To determine this optimal sampling frequency, we developed a general mathematical model for the introduction of a new pathogen that, once introduced, increases in prevalence exponentially. Given the relative cost of infection *vs.* sampling, we derived equations for the expected combined cost per unit time of disease burden and surveillance for a specified sampling frequency, and thus the sampling frequency for which the expected total cost per unit time is lowest.

## 1. Introduction

A key goal of public health infectious disease surveillance systems is to detect a pathogen at an early stage of its entry into the population, enabling interventions to limit its spread and the harm it could inflict (Murray and Cohen, 2017; Budd et al., 2020; Jernigan et al., 2023). Such efforts are increasingly important given the many ways in which communities are connected, with growing populations, global travel, and urbanization, and given ecological shifts associated with climate change and other factors leading to emergence and re-emergence of vector-borne diseases, with cases of locally acquired dengue and malaria where they had been absent for many decades (Baker et al., 2021; Kretschmer et al., 2023; CDC, 2023).

One important strategy for achieving early pathogen detection is monitoring for infected individuals through robust clinical surveillance. However, clinical surveillance is inherently limited in important ways. Infections may be mildly symptomatic, asymptomatic, or have a long pre-symptomatic infectious phase, such that the pathogen population has spread extensively before the first clinical cases are diagnosed and the pathogen is identified (Oran and Topol, 2020). In contexts where access to care or resources is limited, missed cases and reporting delays can make it difficult to rapidly detect and correctly diagnose new infections (Quinn and Kumar, 2014).

For pathogens that can be detected in environmental samples and that spread by vectors, a complementary and critical strategy for early pathogen detection is monitoring through periodic sampling of the environment. Pathogen detection in wastewater has been important for the surveillance and control of poliovirus (Diamond et al., 2022; Shah et al., 2022) and has been used more recently for tracking the local epidemic dynamics and evolution of SARS-CoV-2 (Peccia et al., 2020; Levy et al., 2023), norovirus (Huang et al., 2022), influenza (Mercier et al., 2022), mpox (Chen and Bibby, 2022; Tiwari et al., 2023), and other pathogens (Boehm et al., 2023). Efforts are underway to extend these techniques for tracking antibiotic resistance genes in wastewater (Nguyen et al., 2021; Tiwari et al., 2022). For vector-borne pathogens, including West Nile virus (Petersen et al., 2013), *Borrelia* species (Eisen and Paddock, 2021), and Powassan virus (Hermance and Thangamani, 2017), surveillance includes pathogen detection in vectors collected via traps, with sampling also taking place at a given frequency.

**Fig. 1.**
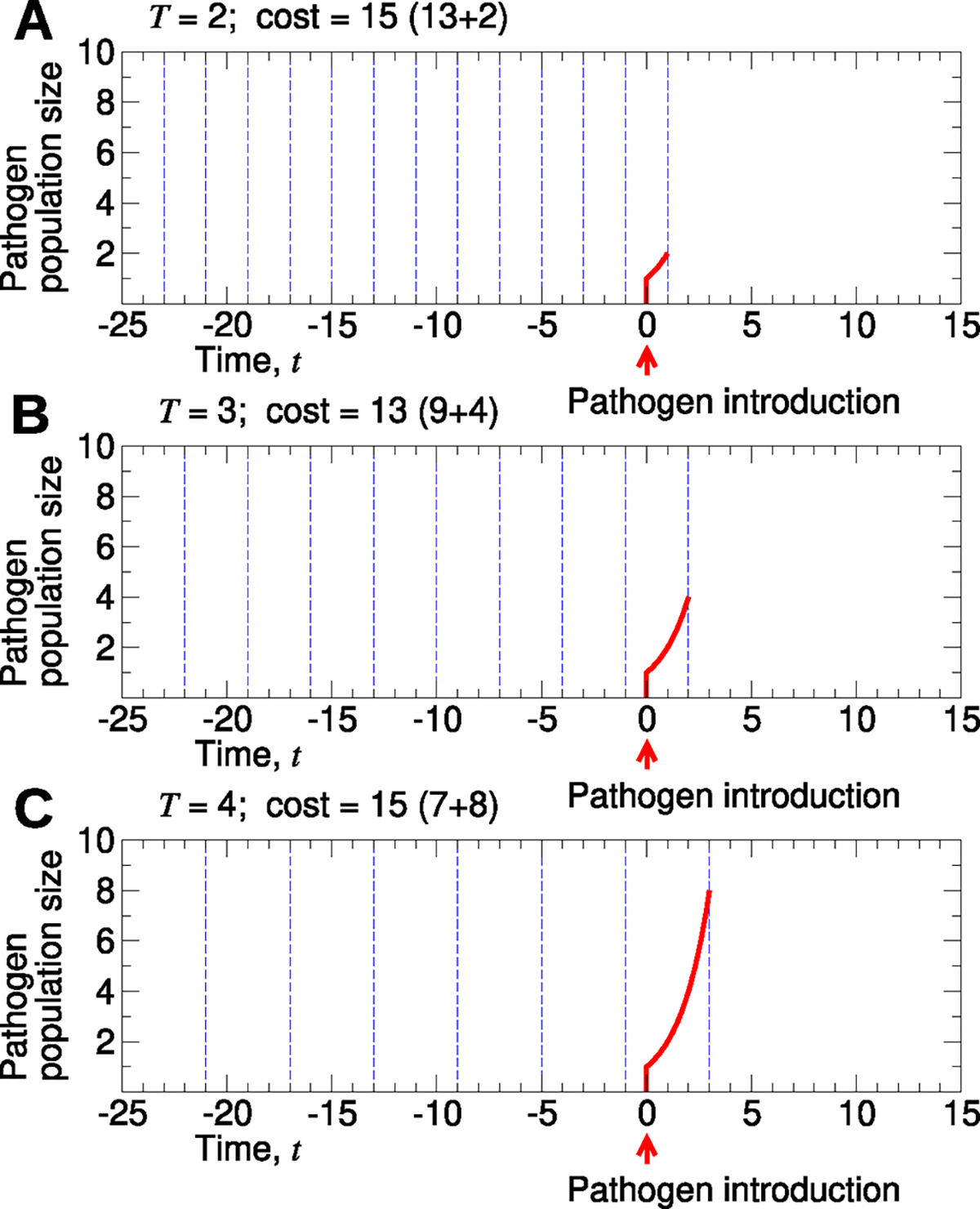
Optimization of surveillance. A simple schematic illustrates how surveillance can be optimized. In the plot, the dotted blue lines represent sampling events, and the solid red curves represent the abundance of a pathogen. Here, we assume that a pathogen first emerges at time t=0 with the pathogen population growing exponentially, doubling at each subsequent time step. Sampling of the environment occurs at times -25+mT – where T is the sampling period and m≥1 is an integer – until the pathogen is first detected. We plot the outcomes if the sampling period had been (**A**) T=2, (**B**) T=3, or (**C**) T=4. If the cost associated with one sampling event is equal to the cost associated with one instance of the pathogen, and if costs accumulate linearly, then T=3 would have resulted in the lowest total cost.

Monitoring for infectious diseases requires substantial time, money, and infrastructure for detection, interpretation, and response (Pfaller, 2001; Vazquez-Prokopec et al., 2010; Braks et al., 2014; Kantor et al., 2022; Ngwira et al., 2022; Hagedorn et al., 2023). Although environmental and vector-based surveillance systems have been recognized and widely discussed for their potential, and despite the massive push to fund and develop these programs—particularly wastewater efforts (Gwinn et al., 2017; Kirby et al., 2021), there remains a critical gap in our understanding: How should surveillance be designed to achieve maximal effectiveness (Gu et al., 2008; Thompson and Etter, 2015; Fournet et al., 2018; Ahmed et al., 2020; Michael-Kordatou et al., 2020; Keshaviah et al., 2021)? A central consideration is how often testing should be performed (Fig. 1). Here, we addressed this question by formulating a simple, stochastic model for pathogen introduction, growth, and detection in the presence of periodic sampling and testing. We identified the key parameters of this process, and we derived a simple equation for the expected total cost per unit time (i.e., the sum of all costs related to surveillance and to effects from the disease divided by the time elapsed, when the stochastic dynamics are run for an arbitrarily long time). The expected total cost per unit time is a function of the parameters of the model, and given values for these parameters, we can minimize this quantity.

**Fig. 2.**
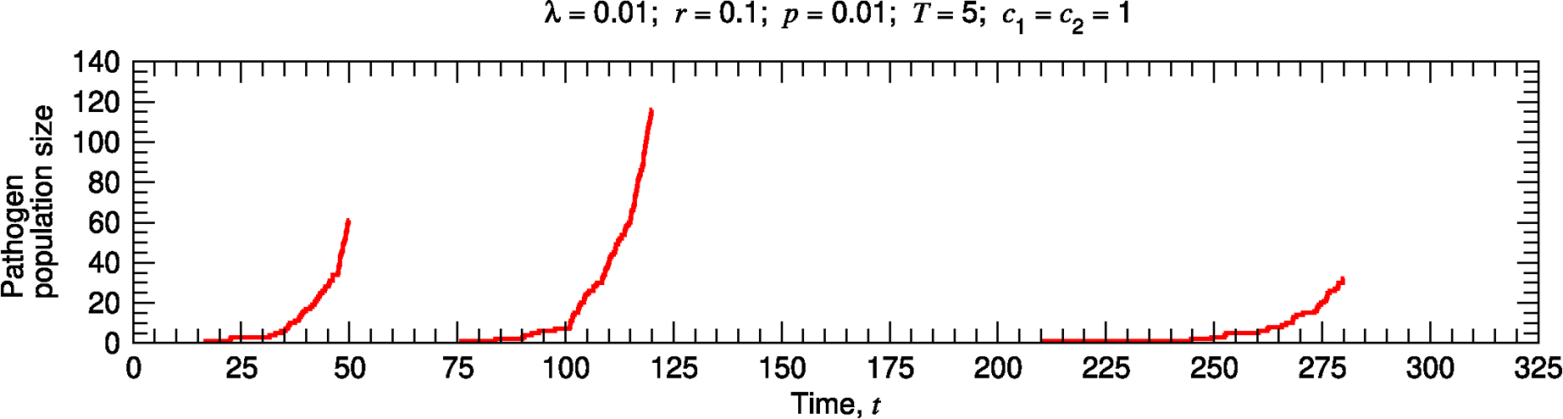
Stochastic surveillance model. A single realization of the stochastic surveillance and disease dynamics is shown. The environment is tested at times 5m, where m is an integer and 1≤m≤65. There are thus 65 testing events, so the cumulative surveillance cost is 65. The first lineage begins at time t≈16.7 and is detected at time t=50, when its size is 61. The second lineage begins at time t≈75.8 and is detected at time t=120, when its size is 116. The third lineage begins at time t≈210.6 and is detected at time t=280, when its size is 32. The cumulative disease cost is thus 61 + 116 + 32 = 209. The total surveillance and disease cost is 65 + 209 = 274, and the total cost per unit time is 274∕325.

Our goal was to minimize the expected total surveillance and disease cost per unit time for the detection of the first appearance of a pathogen. Accordingly, we employed a simple model of surveillance to detect the entry of a pathogen into a population, assuming that, once the pathogen is present, its prevalence increases exponentially. The pathogen can be introduced beginning at time t=0. Sampling begins at time t=T and continues regularly at times $t_{m}=mT$, where m≥1 and T is the sampling period. Each sampling event incurs a cost $c_{1}$, and since there are 1/T sampling events per unit time, the sampling cost per unit time is given by $c_{1}/T$. Copies of the pathogen are introduced after time t=0 according to a Poisson process, such that the waiting times between initiation events are exponentially distributed with rate λ. Once a new lineage is introduced, it also reproduces (transmits) according to a Poisson process, such that the expected prevalence of the pathogen grows exponentially with rate r. If a sampling event detects a copy of a pathogen that belongs to a particular lineage, then that lineage is “detected”. Let p be the probability that a sampling event detects a copy of a pathogen, and since each detection occurs independently, the probability that a sampling event detects a lineage of size n is given by $1-(1-p{)}^{n}$. Once a lineage is detected, we assume that intervention is immediate and is successful at suppressing further spread of that lineage. If a lineage has n copies of the pathogen when it is detected, then the disease cost due to that lineage is given by $c_{2}n$. Letting ⟨n⟩ denote the expected size of a lineage when it is detected, the expected disease cost due to a lineage is given by $c_{2}\langle n\rangle$. Since new lineages appear at rate λ, the expected disease cost per unit time is given by $\lambda c_{2}\langle n\rangle$. The expected total cost per unit time is then $c_{1}/T+\lambda c_{2}\langle n\rangle$. The model is illustrated in Fig. 2.

## 2. Results

We derived an accurate approximation for the expected total cost of testing and disease burden per unit time, C:

(1) $$C=\frac{c_{1}}{T}+\lambda c_{2}\left(\frac{e^{rT}\;-\;1}{rT}\right)\left(1\;-\;\frac{1\;-\;e^{-rT}}{log(1\;-\;p)}\right)$$

Details on the derivation of Eq. (1) are provided in the Supplementary Information. By comparing Eq. (1) with $c_{1}/T+\lambda c_{2}\langle n\rangle$, notice that the product of the two factors in large parentheses is (approximately) equal to the expected size of an outbreak when it is detected, ⟨n⟩. It is helpful to understand the behavior of ⟨n⟩ as a function of p,r, and T. Notice that ⟨n⟩ is a decreasing function of p, an increasing function of r, and an increasing function of T—i.e., a less-sensitive detector, a more rapidly growing pathogen, or a larger testing interval all result in a larger expected size of the outbreak when it is detected. (For very large values of rT, the approximation of Eq. (1) breaks down because the pathogen is typically detected on the first test following its introduction, irrespective of modest changes in p. But such extreme values of rT are unrealistic.)

**Fig. 3.**
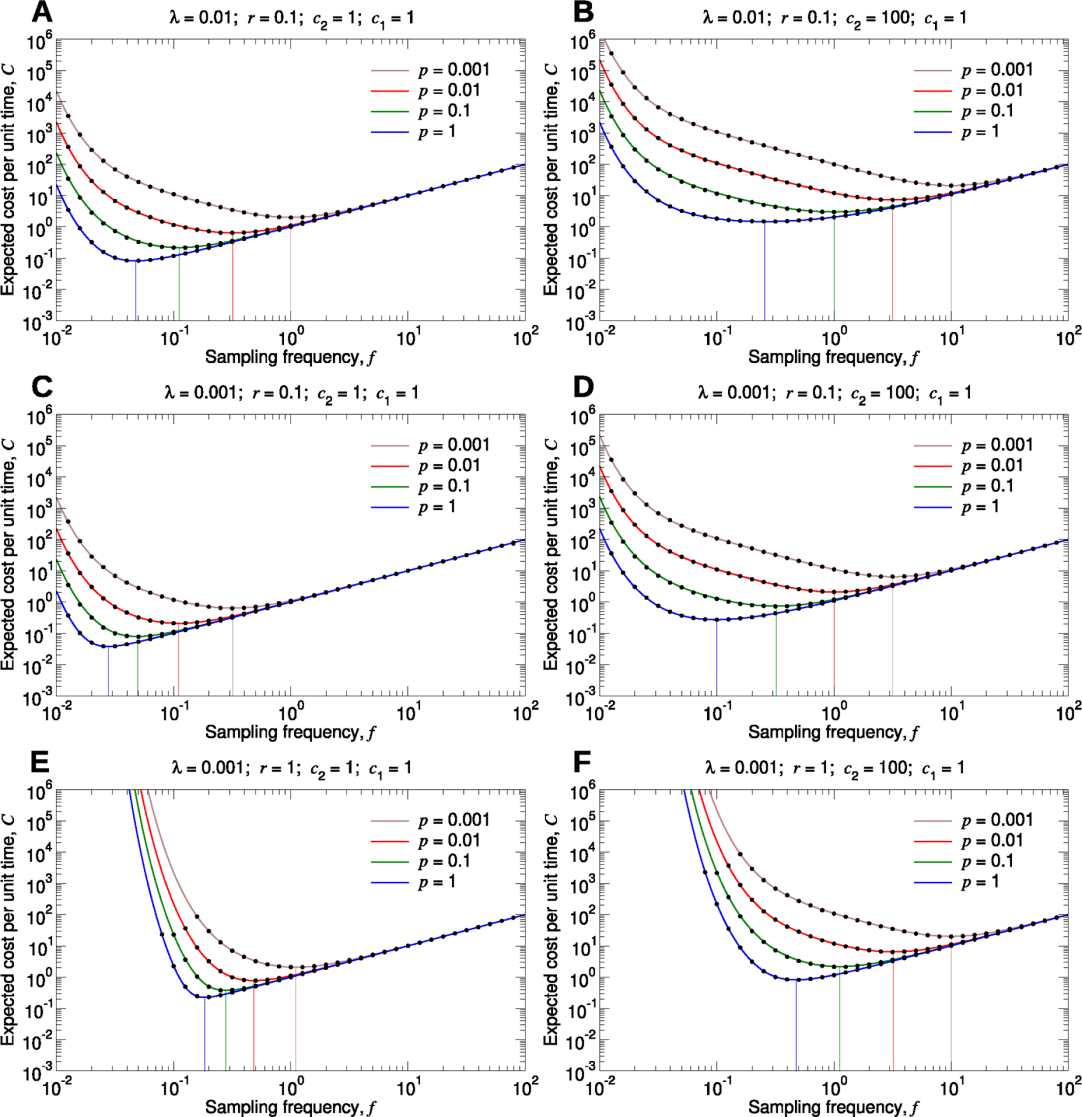
Expected total cost per unit time for a particular type of pathogen. (**A** through **F**) We set $c_{1}=1$, and we plot C, given by Eq. (1), as a function of f for several values of $\lambda ,\;r,\;c_{2}$, and p. The black dots are measurements of the expected total cost per unit time from simulating the true stochastic process. The vertical lines show the sampling frequencies for which this quantity is minimal in each case. For each parameter set, we simulated 10^3^ pathogen introductions, and we computed the total surveillance and pathogen cost divided by the time elapsed. We repeated this simulation sixteen times and calculated the average. Standard errors are smaller than the size of the data points.

The expected infection cost per unit time, $\lambda c_{2}\langle n\rangle$, is therefore also an increasing function of the testing period, T, and this quantity becomes arbitrarily large as T→∞. The surveillance cost per unit time, $c_{1}/T$, however, is a decreasing function of T, and this quantity becomes arbitrarily large as T→0. These behaviors are evident in Fig. 3, where we plotted C as a function of the sampling frequency, f=1/T, for different values of the model parameters. It is instructive to consider the effects of very large or very small values of f on C. As we increase f, we can detect the disease more rapidly, thereby mitigating disease-related costs. But returns are diminishing: We can – at best – hope to discover the disease as a single unit, representing the pathogen introduction, before it has begun to spread and proliferate, while increasing f further can add arbitrarily large surveillance costs. At the other extreme, setting f too small allows the disease to proliferate before any intervention is applied. Therefore, as shown in each of the curves in Fig. 3, C attains a minimum for a particular value of f.

In designing and performing environmental surveillance, we do not know *a priori* the characteristics of a particular pathogen that may be introduced and result in an outbreak. Rather, for optimizing surveillance, the requirement is to have an understanding of the likely characteristics of new pathogens that might emerge. As a simple example, suppose that our surveillance platform is capable of detecting not just one but two different pathogens. Further, suppose that these two pathogens have different costs and are initiated at different rates. Let $c_{2}(1)$ denote the per-case cost for the first pathogen, and let $c_{2}(2)$ denote the per-case cost for the second pathogen. Similarly, let λ(1) denote the rate of introductions for the first pathogen, and let λ(2) denote the rate of introductions for the second pathogen. For this scenario, the expected infection cost per unit time is equal to $\left[\lambda (1)]\left[c_{2}(1)\right][\langle n\rangle ]+[\lambda (2)]\left[c_{2}(2)\right][\langle n\rangle \right]$. It is also possible that the two pathogens differ in their growth rates and in their susceptibility to being detected. The first pathogen might have corresponding parameters r(1) and p(1), while the second pathogen might have parameters r(2) and p(2). As a result, the expected size of an outbreak of the first pathogen, ⟨n⟩(1), might be different from the expected size of an outbreak of the second pathogen, ⟨n⟩(2). The expected infection cost per unit time is then equal to $\left[\lambda (1)]\left[c_{2}(1)\right][\langle n\rangle (1)]+[\lambda (2)]\left[c_{2}(2)\right][\langle n\rangle (2)\right]$. If there are more than two types of pathogens that can emerge and be detected by our surveillance platform, then in the calculation of the expected infection cost per unit time, we would simply add another term for each additional pathogen.

An important point is that the possible values of the parameters $c_{2},\;r$, and p that a pathogen can have are not discrete. Accordingly, let $dc_{2}\;dr\;dp\lambda^{\prime }\left(c_{2},r,p\right)$ denote the (infinitesimal) rate at which pathogens with per-case cost $c_{2}$, growth rate r, and detection probability p emerge. In this more general treatment, $\lambda^{\prime }\left(c_{2},r,p\right)$ is a rate density that is a function of $c_{2},\;r$, and p. To calculate the expected pathogen cost, we integrate $dc_{2}\;dr\;dp\lambda^{\prime }\left(c_{2},r,p\right)c_{2}\langle n\rangle$ over all possible values of $c_{2},\;r$, and p. Accounting for all possible types of pathogens that might emerge, the expected total cost per unit time, $C^{\prime }$, is equal to

(2) $$C^{\prime }=\frac{c_{1}}{T}+\int_{0}^{\infty }\;dc_{2}\int_{0}^{\infty }\;dr\int_{0}^{1}\;\;dp\;\;\times \left\{\lambda^{\prime }\left(c_{2},r,p\right)\left[c_{2}\left(\frac{e^{rT}\;-\;1}{rT}\right)\left(1\;-\;\frac{1\;-\;e^{-rT}}{log(1\;-\;p)}\right)\right]\right\}$$

Eq. (2) can be quickly calculated numerically for different values of the testing period, T. The value of 1/T for which the expected total cost per unit time is lowest specifies the optimal testing frequency, $F^{*}$. From Eq. (2), we have

(3) $$F^{*}=\frac{1}{arg\;{min}_{T}\;C^{\prime }}$$

**Fig. 4.**
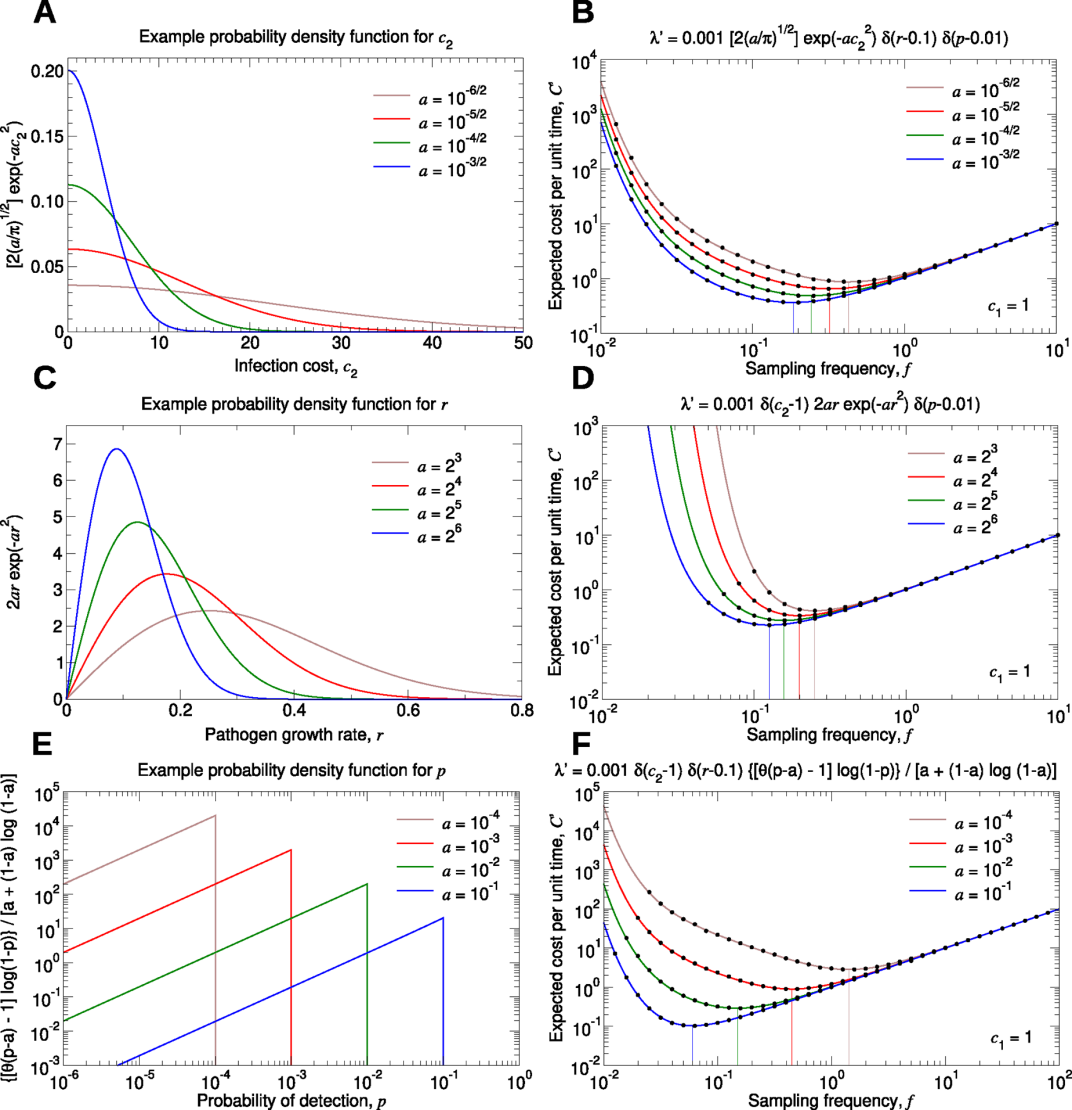
Expected total cost per unit time accounting for many types of pathogens. (**A**, **C**, and **E**) We show example probability density functions for the parameters $c_{2},\;r$, and p, respectively. For each case, we introduce a single parameter, a, which controls the shape of the probability density function. (**B**, **D**, and **F**) We set $c_{1}=1$, and we plot $C^{\prime }$, given by Eq. (2), as a function of f for several rate density functions, $\lambda^{\prime }\left(c_{2},r,p\right)$. The vertical lines show the sampling frequencies for which this quantity is minimal in each case. For each parameter set in (**B**) and (**F**), we simulated 10^3^ pathogen introductions, and for each parameter set in (**D**), we simulated 10^5^ pathogen introductions. For each case, we computed the total surveillance and pathogen cost divided by the time elapsed. We repeated this simulation sixteen times and calculated the average (black dots). Standard errors are smaller than the size of the data points.

Fig. 4 depicts how this works. In Fig. 4A, we show one possible form for the probability density function for $c_{2}$. Pathogens with little or no associated cost (i.e., those for which $c_{2}$ is close to zero) are most common, while more harmful pathogens occasionally arise. The parameter a controls the shape of the probability density function. For smaller values of a, the distribution has a longer tail, meaning that there is a higher chance that a new pathogen is harmful. In Fig. 4B, we use this form for the probability density function for $c_{2}$, we set r=0.1 and p=0.01, we set the total rate of emergence of new pathogens to 0.001, and we plot the expected total cost per unit time, $C^{\prime }$. (In the specification of $\lambda^{\prime },\;\delta$ denotes the Dirac delta function.) For smaller values of a, the optimal testing frequency for environmental surveillance increases accordingly.

Similarly, in Fig. 4C, we show one possible form for the probability density function for r. We again use the parameter a to control the shape of the probability density function. Smaller values of a result in a longer tail to the distribution, so that pathogens with more rapid growth rates are more likely to arise. In Fig. 4D, we use this form for the probability density function for r, we set $c_{2}=1$ and p=0.01, we set the total rate of emergence of new pathogens to 0.001, and we plot $C^{\prime }$. For smaller values of a, we must sample the environment more frequently. Notice that as the sampling frequency decreases below its optimum, the expected total cost per unit time rapidly increases. This is because there is a chance that pathogens with unusually large growth rates are introduced, and if their subsequent exponential growth is not halted soon enough, then the resulting pathogen-associated costs can become extremely large.

In Fig. 4E, we show one possibility for the probability density function for p. For smaller values of a, there is a higher chance of pathogens being introduced that have low sensitivity to being detected. Using this form for the probability density function for p in Fig. 4F, setting $c_{2}=1$ and r=0.1, and setting the total rate of emergence of new pathogens to 0.001, we plot $C^{\prime }$. (In the specification of $\lambda^{\prime },\;\theta$ denotes the Heaviside step function.) Smaller values of a result in a larger optimal testing frequency. Details on Fig. 4 are provided in the Supplementary Information.

The example probability density functions in Fig. 4 were chosen here for convenience: they have nice analytical forms, and they admit simple analytical solutions when substituted into Eq. (2). For optimizing an environmental or vector surveillance system in practice, one would construct an estimated form for $\lambda^{\prime }\left(c_{2},r,p\right)$ based on experimental or observational data, and the optimal testing frequency would be determined numerically using Eq. (3). Optimization of environmental or vector surveillance thus requires an understanding of the cost of each sampling and testing event, $c_{1}$, and an understanding of the function for the rate of emergence of new pathogens, $\lambda^{\prime }\left(c_{2},r,p\right)$.

Estimation of $c_{1}$ and $\lambda^{\prime }\left(c_{2},r,p\right)$ in the context of any surveillance program and set of pathogens would be highly approximate, at best. For applying this theory, it is therefore desirable to have a simple, explicit approximation for the optimal sampling frequency, $F^{*}$. Using Eqs. (2) and (3), we find the following approximation:

(4) $$F^{*}\approx \sqrt{\int_{0}^{\infty }\;dc_{2}\int_{0}^{\infty }\;dr\int_{0}^{1}\;\;dp\left(\frac{c_{2}r}{c_{1}p}\right)\lambda^{\prime }\left(c_{2},r,p\right)}$$

Details on the derivation of Eq. (4) are provided in the Supplementary Information. This approximation for the optimal testing frequency admits a simple understanding. For a particular type of pathogen, $F^{*}$ is an increasing function of $c_{2}$ and r and a decreasing function of $c_{1}$ and p, hence the factor $c_{2}r/\left(c_{1}p\right)$. We then multiply by $\lambda^{\prime }\left(c_{2},r,p\right)\;dc_{2}\;dr\;dp$ and integrate over all possible values of the pathogen-specific parameters. Finally, for the resulting quantity to have dimensions of frequency, we take the square root.

## 3. Discussion

Eqs. (2) and (3) specify the optimal frequency at which to perform sampling and testing. Their use for optimizing testing frequency requires an estimation of the total rate of emergence of new pathogens, $\int_{0}^{\infty }\;dc_{2}\int_{0}^{\infty }\;dr\int_{0}^{1}\;\;dp\lambda^{\prime }\left(c_{2},r,p\right)$, and the likely values of the per-case cost, $c_{2}$, rate of growth, r, and susceptibility to detection, p, of any emerging pathogens. The rate density, $\lambda^{\prime }\left(c_{2},r,p\right)$, is large if pathogens with those parameter values are likely to emerge, and small otherwise. Estimating the dependence of $\lambda^{\prime }$ on p entails many considerations. Molecular properties of emerging pathogens must be anticipated, and they must be interpreted in the context of whichever laboratory tests are used for detection. Spatial structure of the landscape over which pathogens can emerge further influences the dependence of $\lambda^{\prime }$ on p. For instance, if a pathogen emerges far from a wastewater treatment facility, then the number of infections in the vicinity of the location of sampling might be much smaller than the total size of the outbreak. A similar consideration arises in sampling a vector population, where a pathogen might originate and begin spreading in individuals that are far from the nearest trap. Inferring the total rate of emergence of new pathogens and the dependence of $\lambda^{\prime }$ on r may be accomplished by analysis of historical data of either clinical cases or abundance of a pathogen in a vector species, together with maximum likelihood estimation. Optimization of testing frequency further requires a formal understanding of surveillance-related and pathogen-related costs (Zinsstag et al., 2020; Bernstein et al., 2022; Weinstein et al., 2009). Mathematically, the question of how to optimize a surveillance platform is undefined unless all relevant surveillance-related and pathogen-related costs are quantified in the same units. This is challenging, since the underlying factors are inherently very different in nature. Nonetheless, such understanding is essential if environmental and vector surveillance for infectious diseases is to be meaningfully optimized.

Although accurate estimation of these effects and how they influence $c_{1}$ and $\lambda^{\prime }\left(c_{2},r,p\right)$ is challenging, our theory for optimizing the sampling frequency is fairly robust to uncertainties in these quantities. This is evident in the approximation for the optimal sampling frequency given by Eq. (4), where all quantities appear under the square root. In our estimation of $c_{1}$, we could be off by a factor of k (i.e., $c_{1}\to kc_{1}$), but our determination of $F^{*}$ would only be off by a factor of $1/\sqrt{k}$. Likewise, our estimation of $c_{2}$ or r could be off by a factor of k (i.e., $\lambda^{\prime }\left(c_{2},r,p\right)\to \lambda^{\prime }\left(c_{2}/k,r,p\right)$ or $\lambda^{\prime }\left(c_{2},r,p\right)\to \lambda^{\prime }\left(c_{2},r/k,p\right)$ ), but our determination of $F^{*}$ would only be off by a factor of $\sqrt{k}$ in each case.

**Fig. 5.**
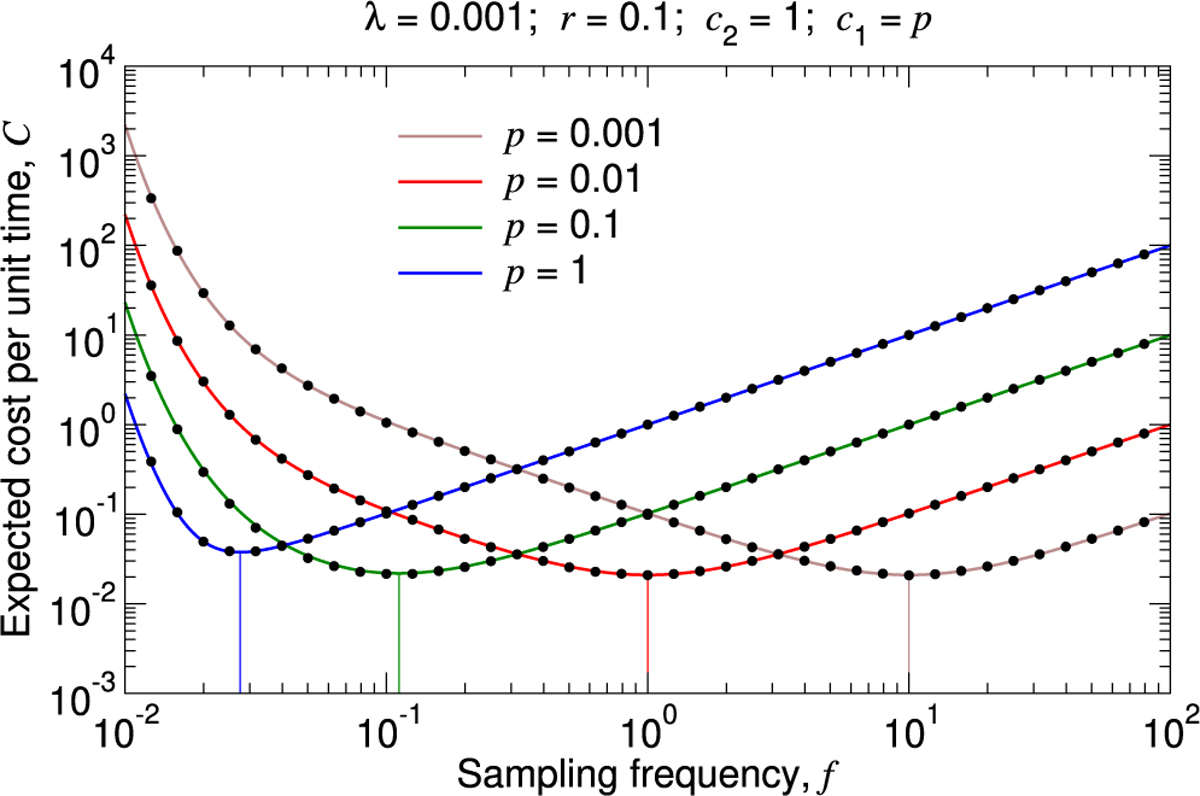
Expected total cost per unit time assuming that surveillance cost depends on sensitivity. We set $c_{1}=p$, and we plot Eq. (1) together with measurements of the expected total cost per unit time from simulating the true stochastic process (black dots). If $c_{1}$ is positively associated with p, then the optimal sampling frequency has a strong inverse relation with p. The vertical lines show the sampling frequencies for which the expected total cost per unit time is minimal in each case. For each parameter set, we simulated 10^3^ pathogen introductions, and we computed the total surveillance and pathogen cost divided by the time elapsed. We repeated this simulation sixteen times and calculated the average. Standard errors are smaller than the size of the data points.

Our model for determining the optimal testing frequency is broadly applicable. The sampling cost, $c_{1}$, is a characteristic of the surveillance platform that is constructed and deployed. $c_{1}$ encompasses many considerations. For a larger population that is more difficult to survey, for example, $c_{1}$ might take a larger value. More precisely, $c_{1}$ is related to both the sensitivity and specificity of the surveillance program. If we consider that specificity is fixed, a more sensitive surveillance program (corresponding to a larger value of p) would likely be more complex and lead to a larger value of $c_{1}$ (Fig. 5). Since the optimal sampling frequency is a decreasing function of both p and $c_{1}$, assuming that $c_{1}$ is directly associated with p results in the optimal sampling frequency having a stronger inverse relationship with p.

If we consider that sensitivity is fixed, the effect of specificity on $c_{1}$, however, is more complex. A more specific surveillance program might be more sophisticated and lead to a larger value of $c_{1}$. But greater specificity also means a lower rate of false positives, resulting in fewer unnecessary intervention costs and effecting a lower value of $c_{1}$. The net effect of specificity on $c_{1}$ for a particular surveillance program and for an estimated set of pathogen characteristics would therefore have to be carefully evaluated.

**Fig. 6.**
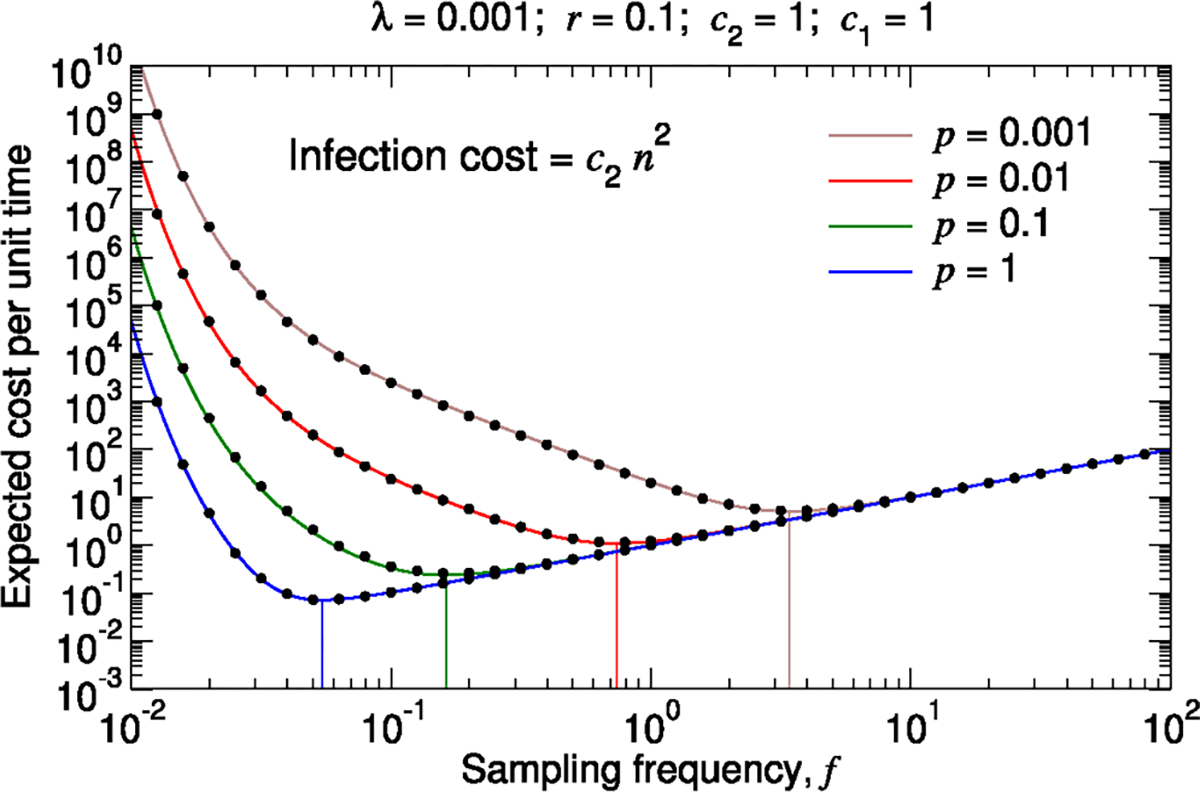
Expected total cost per unit time assuming that the cost due to a single outbreak scales quadratically with the number of clinical cases. We assume that the cost due to a single outbreak equals $c_{2}n^{2}$. We plot Equation S33 in the Supplementary Information together with measurements of the expected total cost per unit time from simulating the true stochastic process (black dots). If the cost due to an outbreak scales quadratically with the number of clinical cases as opposed to linearly, then the optimal sampling frequency is higher. The vertical lines show the sampling frequencies for which the expected total cost per unit time is minimal in each case. For each parameter set, we simulated 10^3^ pathogen introductions, and we computed the total surveillance and pathogen cost divided by the time elapsed. We repeated this simulation sixteen times and calculated the average. Standard errors are smaller than the size of the data points.

Although the long-time dynamics of an emerging pathogen can show complex behavior, the early-time dynamics are often approximately exponential, and the associated disease-related costs at early times are expected to scale roughly linearly with the size of the outbreak. Both of these features are incorporated in our model. Nonetheless, our framework can handle alternative assumptions. For example, in Fig. 6, we show the expected total cost per unit time if the cost of a single outbreak is equal to $c_{2}n^{2}$. For low sampling frequencies, the expected total cost per unit time is larger than for the case where pathogen costs scale linearly with outbreak size. Accordingly, the optimal sampling frequency is increased.

**Fig. 7.**
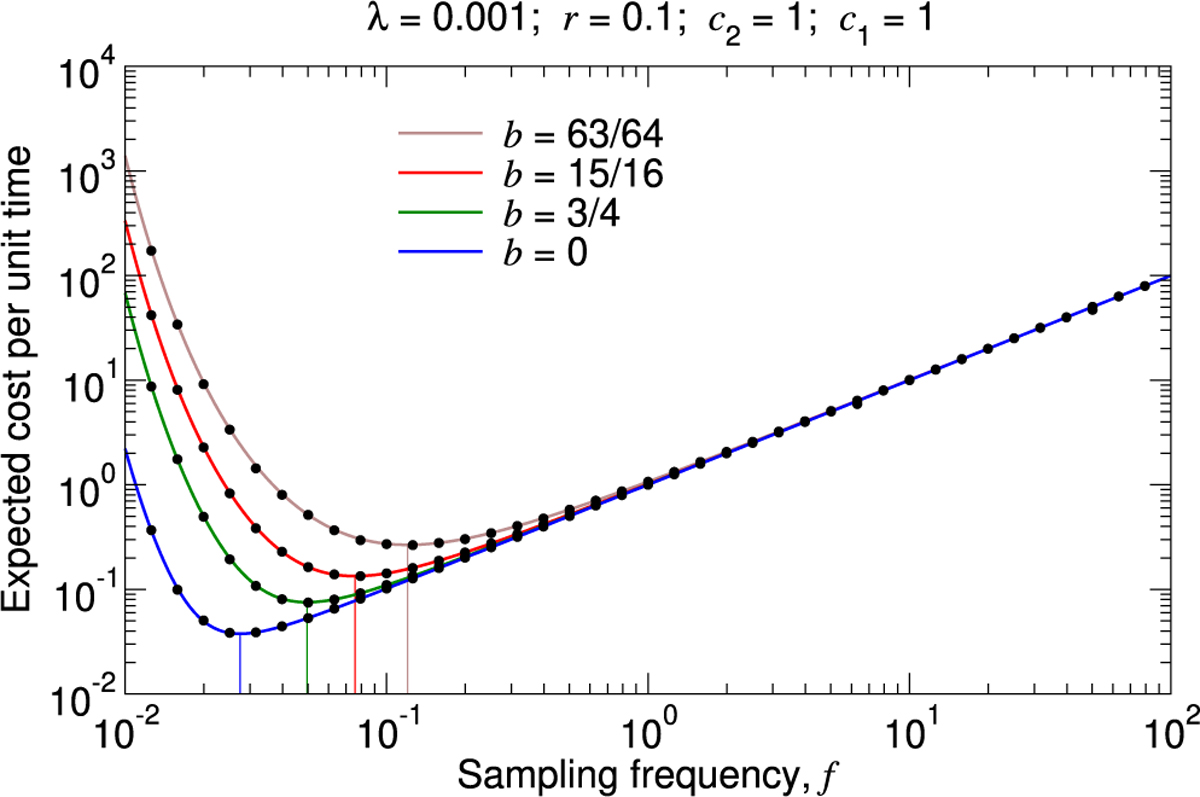
Expected total cost per unit time considering the possibility of breakthrough infections. We plot Equation S39 in the Supplementary Information together with measurements of the expected total cost per unit time from simulating the true stochastic process (black dots). Whenever an outbreak is detected and controlled, with probability b, there is a single new infection that initiates immediately thereafter. For larger values of b, the optimal sampling frequency is higher. The vertical lines show the sampling frequencies for which the expected total cost per unit time is minimal in each case. For each parameter set, we simulated 10^3^ pathogen introductions, and we computed the total surveillance and pathogen cost divided by the time elapsed. We repeated this simulation sixteen times and calculated the average. Standard errors are smaller than the size of the data points.

Once a pathogen is detected and an intervention is implemented, spread of the pathogen and its associated costs are not immediately halted, and this can be approximately accounted for by making the substitution $\lambda^{\prime }\left(c_{2},r,p\right)\to \lambda^{\prime }\left(c_{2}/k,r,p\right)$, where k>1. A further consideration is that intervention is unlikely to completely eliminate the pathogen. Subsequent sampling and testing would then monitor for when the pathogen becomes sufficiently abundant again that additional intervention is warranted. This may be approximately described by using a rate density for introductions that is time-dependent (i.e., $\lambda^{\prime }\left(c_{2},r,p\right)\to \lambda^{\prime }\left(c_{2},r,p,t\right)$) and increases if there was a recently suppressed outbreak. The increased value of $\lambda^{\prime }$ accounts for the possibility of a follow-up outbreak due to cases that the intervention failed to extinguish. As a simple example, in Fig. 7, we show simulations of a slightly generalized model in which breakthrough infections are possible. Whenever a lineage is detected, all cases are controlled, but one new infection is initiated immediately following the intervention with probability b. (Conversely, with probability 1-b, there is no breakthrough infection.) If there is a breakthrough infection, then that infection multiplies, and its lineage must similarly be detected and controlled. For values of b>0, infections that escape control must be quickly detected and halted, so the optimal testing frequency increases.

Changes in weather and climate affect the risk of an outbreak – especially for many vector-borne pathogens (Pley et al., 2021) – and this could also be modeled through time-dependence of $\lambda^{\prime }$. A key observation is that sampling is usually done on a timescale of days or weeks, whereas seasonality is typically related to the time of year. With this separation of timescales, Eqs. (2) and (3) approximately specify the optimal sampling frequency at any given time of year. We treat $\lambda^{\prime }$ as being periodic with period equal to one year, and we substitute this time-dependent rate density into Eq. (2).

Our approach can be applied to answer another pressing question: Where should environmental sampling be performed? A pathogen may be more likely to emerge in certain locations than others, and certain parts of the population may be more difficult to survey than others. To address these points, we can include a spatial structure in the model. The pathogen can be introduced in one location and then migrate to different locations as it proliferates. By numerically running the stochastic dynamics with spatial structure, an expected total surveillance and disease cost per unit time can be calculated. By trying different sampling locations, it is possible to find the sampling locations for which this quantity is minimal.

Environmental and vector surveillance are equally instrumental for tracking the prevalence of a pathogen (Teklehaimanot et al., 2004). An understanding of how and when to intervene is therefore essential (Lipsitch et al., 2009; Peak et al., 2017). If false positives are too frequent, then intervention costs will accumulate, leading to costly surveillance. If the designated signal that is required for intervention is too strong, then the pathogen can spread to the point where intervention has limited effectiveness in mitigating disease-related costs. The optimal testing frequency could also be adjusted as new data become available (DeFelice et al., 2017). If tracking indicates increased prevalence of a pathogen, then more frequent sampling and testing might be warranted. For example, for the model simulated in Fig. 7, instead of having a constant sampling frequency, we could increase the sampling frequency immediately following detection of a lineage to guard against the possibility of an infection that evades control. If there are no positive tests for a certain time afterward, then the sampling frequency might be safely lowered to its original value. For seasonal infections, a further possibility is that testing could be performed frequently for several years to gain an understanding of the typical seasonal behavior for new pathogens or in new ecological settings. This could inform optimization and enable more efficient tracking of the pathogen’s abundance.

In practice, a surveillance program would be executed over a defined time period, where the stochasticity in the origination and growth of new infections is of paramount concern. It is possible that for many realizations of the dynamics, no infections emerge, and the total cost consists only of costs related to surveillance. Other realizations might be characterized by emergence of just one or two pathogens that inflict substantial harm. The expected total cost per unit time over an arbitrarily long time interval might therefore not be the best metric for optimizing testing frequency. An alternative would be to use the rate density for origination of new pathogens, $\lambda^{\prime }$, to estimate the probability of either an unusually large number of pathogens or an uncharacteristically lethal pathogen. To mitigate the possibility of excessive harm from these rare events, the optimal testing frequency could be increased appropriately.

Our model and its many possible extensions can inform the design of these critical aspects of environmental and vector surveillance platforms. Our work provides a general and robust foundation for mechanistic optimization of environmental surveillance for infectious diseases.

## Supplementary Material

1
