# Supplementary material for: Optimal environmental testing frequency for outbreak surveillance
Source: Epidemics. Author manuscript; Available in PMC 2024 Mar 29. (PMC10979539; doi:10.1016/j.epidem.2024.100750)
Supplement: 1 [file NIHMS1973914-supplement-1.pdf]

Supplementary Information: Optimal environmental testing frequency for outbreak surveillance

Jason W. Olejarz, Kirstin I. Oliveira Roster, Stephen M. Kissler,
Marc Lipsitch, Yonatan H. Grad

This Supplementary Information is organized as follows: In Section 1, we describe the surveillance protocol under consideration, and we derive the surveillance cost per unit time. In Section 2, we define the process by which new pathogens emerge, we define the dynamics of a pathogen that is growing in abundance, we define the manner in which each pathogen is detected, and we calculate the expected size of an outbreak. In Section 3, we calculate the expected total cost per unit time, and we determine the optimal testing frequency. In Section 4, we describe how our model generalizes to account for the emergence of pathogens with different characteristics. In Section 5, we derive a simple approximation for the optimal testing frequency. In Section 6, we explore some extensions of our model.

1 Surveillance cost

An important consideration for implementing environmental surveillance for pathogens is the frequency at which tests are performed. Environmental sampling and testing should be done frequently enough that an emerging pathogen is intercepted quickly, but not so frequently that surveillance costs outweigh the benefits of early detection. Here, we assume that whenever the environment is sampled and a test is conducted, a surveillance cost equal to c_1 is incurred. We also assume that surveillance costs are additive, so that if n tests are performed, then the total surveillance cost is equal to nc_1.

We consider that environmental tests are performed with period T. Since the cost of a single test is equal to c_1, and since the time between tests is equal to T, the surveillance cost per unit time is given by

$$C_1 = \frac{c_1}{T} \tag{S1}$$

2 Expected infection cost

The costs incurred from the surveillance program itself must be considered in the context of infection-related costs. The costs due to an outbreak can vary depending on several factors:

- When the pathogen first appears in relation to the first environmental test that is performed following its introduction

- How the pathogen grows after it is introduced
- The sensitivity of the environmental testing program for detecting the pathogen
- The per-case infection cost

In this section, we describe each of these points in detail. Considering the full stochastic dynamics of pathogen initiation, pathogen growth, and pathogen detection, we derive a solution for the expected size of an outbreak when it is detected. We then derive an approximation for the expected size of an outbreak by assuming deterministic growth of a pathogen after it is initiated.

2.1 Emergence of a pathogen

For determining the optimal testing frequency, we require knowledge of how new pathogens are introduced. We assume that the introduction of new pathogens follows a Poisson process. New pathogens are initiated independently and continuously in time at rate λ.

2.2 Growth of a pathogen

We also require knowledge of how a pathogen increases in abundance once it first appears. Here, we assume that each instance of the pathogen makes new instances of the pathogen at rate r according to a Poisson process. Let $x_{m,n}(rt)$ denote the probability that there are n copies of the pathogen at time t, given that there are m copies of the pathogen at time 0. In this section, we present the steps for calculating $x_{m,n}(rt)$, beginning with the simplest cases and then progressing to the solution for any values of m and n, where $m \leq n$.

2.2.1 $m = 1$, $n = 1$

Suppose we start with a single instance of the pathogen at time 0 ($m = 1$). $x_{1,1}(rt)$ gives the probability that the original instance of the pathogen has not produced any new instances of the pathogen up to time t ($n = 1$). $x_{1,1}(rt)$ is given by

$$x_{1,1}(rt) = e^{-rt}$$

2.2.2 $m = 1$, $n = 2$

Next, consider $x_{1,2}(rt)$, which is the probability that the original instance of the pathogen has produced a single new instance of the pathogen by time t ($n = 2$). For this to occur, three things must happen: The original instance of the pathogen does not make any new instances of the pathogen between times 0 and t_1, the original instance of the pathogen makes a new copy of itself at time t_1, and neither of the two resulting instances of the pathogen make any new instances of the pathogen between times t_1 and t. We must integrate over all values of t_1 between 0 and t:

$$x_{1,2}(rt) = \int_{t_1=0}^{t} e^{-rt_1}(r\,\mathrm{d}t_1)e^{-2r(t-t_1)}$$

Simplifying, we have

$$x_{1,2}(rt) = \left(\int_{t_1=0}^{t} e^{rt_1}(r\,\mathrm{d}t_1)\right) e^{-2rt}$$

Performing the integration, we get

$$x_{1,2}(rt) = \left(e^{rt} - 1\right) e^{-2rt}$$

This then becomes

$$x_{1,2}(rt) = \left(1 - e^{-rt}\right) e^{-rt}$$

2.2.3 $m = 1$, $n = 3$

Next, consider $x_{1,3}(rt)$, which is the probability that the original instance of the pathogen has led to two new instances of the pathogen by time t ($n = 3$). For this to occur, five things must happen: The original instance of the pathogen does not make any new instances of the pathogen between times 0 and t_2, the original instance of the pathogen makes a new copy of itself at time t_2, neither of the two resulting instances of the pathogen make any new instances of the pathogen between times t_2 and t_1, one of the two instances of the pathogen makes a new copy of itself at time t_1, and none of the three resulting instances of the pathogen make any new instances of the pathogen between times t_1 and t. We must integrate over all values of t_2 between 0 and t_1, and we must integrate over all values of t_1 between 0 and t:

$$x_{1,3}(rt) = \int_{t_1=0}^{t} \int_{t_2=0}^{t_1} e^{-rt_2}(r\,\mathrm{d}t_2)e^{-2r(t_1-t_2)}(2r\,\mathrm{d}t_1)e^{-3r(t-t_1)}$$

We can extend the range of the integration over t_2 from $t_2 = 0$ to $t_2 = t$ if we also divide by 2:

$$x_{1,3}(rt) = \frac{1}{2}\int_{t_1=0}^{t} \int_{t_2=0}^{t} e^{-rt_2}(r\,\mathrm{d}t_2)e^{-2r(t_1-t_2)}(2r\,\mathrm{d}t_1)e^{-3r(t-t_1)}$$

Simplifying, we have

$$x_{1,3}(rt) = \left(\int_{t_2=0}^{t} e^{rt_2}(r\,\mathrm{d}t_2)\right)\left(\int_{t_1=0}^{t} e^{rt_1}(r\,\mathrm{d}t_1)\right) e^{-3rt}$$

Performing the integration, we get

$$x_{1,3}(rt) = \left(e^{rt} - 1\right)^2 e^{-3rt}$$

This then becomes

$$x_{1,3}(rt) = \left(1 - e^{-rt}\right)^2 e^{-rt}$$

2.2.4 $m = 1$, $n = 4$

Next, consider $x_{1,4}(rt)$, which is the probability that the original instance of the pathogen has led to three new instances of the pathogen by time t ($n = 4$). For this to occur, seven things must happen: The original instance of the pathogen does not make any new instances

of the pathogen between times 0 and t_3, the original instance of the pathogen makes a new copy of itself at time t_3, neither of the two resulting instances of the pathogen make any new instances of the pathogen between times t_3 and t_2, one of the two instances of the pathogen makes a new copy of itself at time t_2, none of the three resulting instances of the pathogen make any new instances of the pathogen between times t_2 and t_1, one of the three instances of the pathogen makes a new copy of itself at time t_1, and none of the four resulting instances of the pathogen make any new instances of the pathogen between times t_1 and t. We must integrate over all values of t_3 between 0 and t_2, we must integrate over all values of t_2 between 0 and t_1, and we must integrate over all values of t_1 between 0 and t:

$$x_{1,4}(rt) = \int_{t_1=0}^{t} \int_{t_2=0}^{t_1} \int_{t_3=0}^{t_2} e^{-rt_3}(r\,\mathrm{d}t_3) e^{-2r(t_2-t_3)}(2r\,\mathrm{d}t_2) e^{-3r(t_1-t_2)}(3r\,\mathrm{d}t_1) e^{-4r(t-t_1)}$$

We can extend the range of the integration over t_3 from $t_3 = 0$ to $t_3 = t$ and the range of the integration over t_2 from $t_2 = 0$ to $t_2 = t$ if we also divide by 3!:

$$x_{1,4}(rt) = \frac{1}{3!} \int_{t_1=0}^{t} \int_{t_2=0}^{t} \int_{t_3=0}^{t} e^{-rt_3}(r\,\mathrm{d}t_3) e^{-2r(t_2-t_3)}(2r\,\mathrm{d}t_2) e^{-3r(t_1-t_2)}(3r\,\mathrm{d}t_1) e^{-4r(t-t_1)}$$

Simplifying, we have

$$x_{1,4}(rt) = \left(\int_{t_3=0}^{t} e^{rt_3}(r\,\mathrm{d}t_3)\right) \left(\int_{t_2=0}^{t} e^{rt_2}(r\,\mathrm{d}t_2)\right) \left(\int_{t_1=0}^{t} e^{rt_1}(r\,\mathrm{d}t_1)\right) e^{-4rt}$$

Performing the integration, we get

$$x_{1,4}(rt) = \left(e^{rt} - 1\right)^3 e^{-4rt}$$

This then becomes

$$x_{1,4}(rt) = \left(1 - e^{-rt}\right)^3 e^{-rt}$$

2.2.5 $m = 1$, any n

We can generalize the calculation to arbitrary values of n:

$$x_{1,n}(rt_0) = \left(\prod_{j=1}^{n-1} \int_{t_j=0}^{t_{j-1}} e^{-(n-j+1)r(t_{j-1}-t_j)}(n-j)(r\,\mathrm{d}t_j)\right) e^{-rt_{n-1}}$$

Changing the integration limits, we have

$$x_{1,n}(rt_0) = \frac{1}{(n-1)!} \left(\prod_{j=1}^{n-1} \int_{t_j=0}^{t_0} e^{-(n-j+1)r(t_{j-1}-t_j)}(n-j)(r\,\mathrm{d}t_j)\right) e^{-rt_{n-1}}$$

This simplifies to

$$x_{1,n}(rt_0) = \left(\prod_{j=1}^{n-1} \int_{t_j=0}^{t_0} e^{-(n-j+1)r(t_{j-1}-t_j)}(r\,\mathrm{d}t_j)\right) e^{-rt_{n-1}}$$

This becomes

$$x_{1,n}(rt_0) = \left(\int_{t=0}^{t_0} e^{rt}(r\,\mathrm{d}t)\right)^{n-1} e^{-nrt_0}$$

Performing the integration, we get

$$x_{1,n}(rt) = \left(1 - e^{-rt}\right)^{n-1} e^{-rt} \tag{S2}$$

2.2.6 Any m and n

Following the same procedure, we can calculate $x_{m,n}(rt)$:

$$x_{m,n}(rt_0) = \left(\prod_{j=1}^{n-m} \int_{t_j=0}^{t_{j-1}} e^{-(n-j+1)r(t_{j-1}-t_j)}(n-j)(r\,\mathrm{d}t_j)\right) e^{-mrt_{n-m}}$$

Changing the integration limits, we have

$$x_{m,n}(rt_0) = \frac{1}{(n-m)!}\left(\prod_{j=1}^{n-m} \int_{t_j=0}^{t_0} e^{-(n-j+1)r(t_{j-1}-t_j)}(n-j)(r\,\mathrm{d}t_j)\right) e^{-mrt_{n-m}}$$

This simplifies further:

$$x_{m,n}(rt_0) = \frac{(n-1)!}{(n-m)!(m-1)!}\left(\prod_{j=1}^{n-m} \int_{t_j=0}^{t_0} e^{-(n-j+1)r(t_{j-1}-t_j)}(r\,\mathrm{d}t_j)\right) e^{-mrt_{n-m}}$$

We can rewrite this as

$$x_{m,n}(rt_0) = \binom{n-1}{m-1}\left(\prod_{j=1}^{n-m} \int_{t_j=0}^{t_0} e^{-(n-j+1)r(t_{j-1}-t_j)}(r\,\mathrm{d}t_j)\right) e^{-mrt_{n-m}}$$

This becomes

$$x_{m,n}(rt_0) = \binom{n-1}{m-1}\left(\int_{t=0}^{t_0} e^{rt}(r\,\mathrm{d}t)\right)^{n-m} e^{-nrt_0}$$

Performing the integration, we get

$$x_{m,n}(rt) = \binom{n-1}{m-1}\left(1 - e^{-rt}\right)^{n-m} e^{-mrt} \tag{S3}$$

2.3 Detection of a pathogen

We further require an understanding of how the outbreak is detected. Consider that there are n instances of the pathogen within a particular lineage when the environment is tested. We assume that each instance of the pathogen is not detected independently with probability q. The outbreak is not detected if and only if no instance of the pathogen is detected, which occurs with probability q^n. Therefore, the pathogen is detected with probability $1 - q^n$.

We further assume that each lineage of the pathogen is detected independently of any other lineage. For example, suppose that two lineages of the pathogen are simultaneously

present. Suppose that when the environment is tested, Lineage A contains n_A copies of the pathogen, and Lineage B contains n_B copies of the pathogen. In this case, Lineage A is detected with probability $1 - q^{n_A}$, and Lineage B is detected with probability $1 - q^{n_B}$. (If the rate of introduction of new pathogens, λ, is small, then simultaneous presence of two lineages would be a rare occurrence. Nonetheless, we describe this possibility so that the stochastic dynamics of pathogen initiation, pathogen growth, and pathogen detection are completely specified.)

2.4 Expected size of an outbreak when it is detected

Using the stochastic rules presented above, and using Equation (S3), we can derive a formula for the expected size of an outbreak when the pathogen is detected. For understanding the steps of the calculation, we define $X_i(a_i)$ to be the probability that there are i testing events following the appearance of the pathogen that fail to detect the pathogen, and that there are a_i infections when the pathogen is detected.

We first consider the following question: What is the probability that the pathogen is detected in the first test following its appearance and that there are a_0 instances of the pathogen when it is detected? This probability, which we denote $X_0(a_0)$, is given by

$$X_0(a_0) = \int_0^T \left(\frac{\mathrm{d}\tau}{T}\right) x_{1,a_0}(r\tau)(1 - q^{a_0})$$

There are three components to this calculation:

- The pathogen is initiated at time τ before the testing event that detects it occurs. If the pathogen emerges just before the test that detects it is performed, then τ is slightly greater than 0. If the pathogen emerges just after the previous test, then τ is slightly less that T. Therefore, we have $0 \leq \tau < T$. Since new lineages appear independently and continuously in time, τ is equiprobably distributed between 0 and T, hence the integration $\int_0^T \mathrm{d}\tau/T$.
- The pathogen begins as a single infection, and it grows to a_0 infections at time τ since its appearance with probability $x_{1,a_0}(r\tau)$.
- At least one of the a_0 infections is detected with probability $1 - q^{a_0}$.

Next, we can ask: What is the probability that the pathogen is detected in the second test following its appearance and that there are a_1 instances of the pathogen when it is detected? This probability, which we denote $X_1(a_1)$, is given by

$$X_1(a_1) = \int_0^T \frac{\mathrm{d}\tau}{T} \sum_{a_0=1}^{a_1} x_{1,a_0}(r\tau) q^{a_0} x_{a_0,a_1}(rT)(1 - q^{a_1})$$

This calculation is understood as follows: The first test occurs at time τ after the pathogen appears, the pathogen grows to a_0 infections at time τ after its emergence, none of those a_0 infections are detected in the first test, the pathogen then grows to a_1 infections at time

$\tau + T$ after its emergence, and at least one of those a_1 infections is detected in the second test. We must sum over all values of a_0 between 1 and a_1.

We can further ask: What is the probability that the pathogen is detected in the third test following its appearance and that there are a_2 instances of the pathogen when it is detected? This probability, which we denote $X_2(a_2)$, is given by

$$X_2(a_2) = \int_0^T \frac{\mathrm{d}\tau}{T} \sum_{a_1=1}^{a_2} \sum_{a_0=1}^{a_1} x_{1,a_0}(r\tau) q^{a_0} x_{a_0,a_1}(rT) q^{a_1} x_{a_1,a_2}(rT)(1-q^{a_2})$$

This calculation is understood as follows: The first test occurs at time τ after the pathogen appears, the pathogen grows to a_0 infections at time τ after its emergence, none of those a_0 infections are detected in the first test, the pathogen then grows to a_1 infections at time $\tau + T$ after its emergence, none of those a_1 infections are detected in the second test, the pathogen then grows to a_2 infections at time $\tau + 2T$ after its emergence, and at least one of those a_2 infections is detected in the third test. We must sum over all values of a_0 between 1 and a_1 and over all values of a_1 between 1 and a_2.

The calculation of $X_3(a_3)$ follows in the same manner:

$$X_3(a_3) = \int_0^T \frac{\mathrm{d}\tau}{T} \sum_{a_2=1}^{a_3} \sum_{a_1=1}^{a_2} \sum_{a_0=1}^{a_1} x_{1,a_0}(r\tau) q^{a_0} x_{a_0,a_1}(rT) q^{a_1} x_{a_1,a_2}(rT) q^{a_2} x_{a_2,a_3}(rT)(1-q^{a_3})$$

To calculate the expected size of an outbreak, we sum $X_m(a_m)a_m$ over all possible sizes of the outbreak when the pathogen is detected ($1 \leq a_m < \infty$) and over all possible numbers of failed tests ($0 \leq m < \infty$):

$$\langle n \rangle = \sum_{m=0}^{\infty} \sum_{a_m=1}^{\infty} X_m(a_m) a_m \tag{S4}$$

Equation (S4) can be written as follows:

$$\langle n \rangle = \int_0^T \frac{\mathrm{d}\tau}{T} \sum_{m=0}^{\infty} \sum_{a_m=1}^{\infty} \left[\sum_{1 \leq a_{j-1} \leq a_j} x_{1,a_0}(r\tau) \left(\prod_{j=1}^{m} q^{a_{j-1}} x_{a_{j-1},a_j}(rT) \right) \right] (1-q^{a_m}) a_m \tag{S5}$$

2.5 Approximation for $\langle n \rangle$

Equation (S5) is analytically unwieldy. To make progress, we derive an approximate solution for the expected size of an outbreak.

2.5.1 $p = 1$

To calculate a solution for $\langle n \rangle$ for $p = 1$, we use Equation (S2), and we integrate over all possible values of τ between 0 and T:

$$\begin{aligned}
\langle n \rangle\big|_{p=1} &= \int_0^T \frac{\mathrm{d}\tau}{T} \sum_{n=1}^{\infty} n x_{1,n}(r\tau) \\
&= \int_0^T \frac{\mathrm{d}\tau}{T} \sum_{n=0}^{\infty} n \left(1 - e^{-r\tau}\right)^{n-1} e^{-r\tau} \\
&= \frac{1}{rT} \int_0^T \mathrm{d}\tau \, \frac{\mathrm{d}}{\mathrm{d}\tau} \sum_{n=0}^{\infty} (1 - e^{-r\tau})^n \\
&= \frac{1}{rT} \int_0^T \mathrm{d}\tau \, \frac{\mathrm{d}}{\mathrm{d}\tau} (e^{r\tau})
\end{aligned}$$

Performing the integration, we obtain

$$\langle n \rangle\big|_{p=1} = \frac{e^{rT} - 1}{rT} \tag{S6}$$

2.5.2 $p \ll 1$ and $rT \ll 1$

To calculate an approximate solution for $\langle n \rangle$ for small values of p, we assume that the pathogen grows deterministically after it is initiated (Figure S1). The first several steps of this process are as follows:

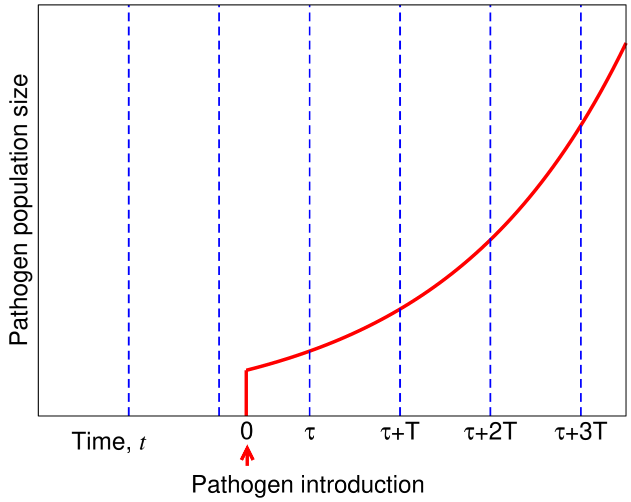

Figure S1: **Schematic showing deterministic growth of the pathogen.** For calculating an approximation for the expected size of an outbreak when it is detected, we can assume that the size of the outbreak grows deterministically.

- The first infection occurs, and at time τ after its emergence, a test is performed. The size of the outbreak when the first test is performed is equal to $e^{r\tau}$.

- If the pathogen is not detected in the first test, which occurs with probability $q^{e^{r\tau}}$, then the pathogen grows until the second test is performed, and the size of the outbreak is equal to $e^{r(\tau+T)}$.
- If the pathogen is not detected in the first test and the second test, which occurs with probability $q^{e^{r\tau}} q^{e^{r(\tau+T)}}$, then the pathogen grows until the third test is performed, and the size of the outbreak is equal to $e^{r(\tau+2T)}$.
- If the pathogen is not detected in the first test, the second test, and the third test, which occurs with probability $q^{e^{r\tau}} q^{e^{r(\tau+T)}} q^{e^{r(\tau+2T)}}$, then the pathogen grows until the fourth test is performed, and the size of the outbreak is equal to $e^{r(\tau+3T)}$.

This process continues until the pathogen is detected. We therefore have the following result for the expected size of an outbreak:

$$\begin{aligned}\langle n\rangle\big|_{p,rT\ll 1} \approx \int_0^T \frac{\mathrm{d}\tau}{T}\Big[&e^{r\tau}\\ &+ q^{e^{r\tau}}\left(e^{r(\tau+T)} - e^{r\tau}\right)\\ &+ q^{e^{r\tau}} q^{e^{r(\tau+T)}}\left(e^{r(\tau+2T)} - e^{r(\tau+T)}\right)\\ &+ q^{e^{r\tau}} q^{e^{r(\tau+T)}} q^{e^{r(\tau+2T)}}\left(e^{r(\tau+3T)} - e^{r(\tau+2T)}\right)\\ &+\cdots\Big]\end{aligned}$$

Considering that $p \ll 1$ and $rT \ll 1$, this can be simplified:

$$\langle n\rangle\big|_{p,rT\ll 1} \approx q^{e^{rT}}\left(e^{2rT} - e^{rT}\right) + q^{e^{rT}} q^{e^{2rT}}\left(e^{3rT} - e^{2rT}\right) + q^{e^{rT}} q^{e^{2rT}} q^{e^{3rT}}\left(e^{4rT} - e^{3rT}\right) + \cdots$$

More compactly:

$$\langle n\rangle\big|_{p,rT\ll 1} \approx \left(e^{rT} - 1\right)\sum_{k=1}^{\infty} e^{krT} q^{e^{krT}\sum_{x=0}^{k-1} e^{-xrT}} \tag{S7}$$

The process can also be considered by defining

$$p \equiv 1 - q \tag{S8}$$

Here, p is the probability that a single infection is detected in a testing event, so that the probability that an outbreak of size n is detected in a testing event is given by $1-(1-p)^n$. Substituting Equation (S8) into Equation (S7), we have

$$\langle n\rangle\big|_{p,rT\ll 1} \approx \left(e^{rT} - 1\right)\sum_{k=1}^{\infty} e^{krT}(1-p)^{e^{krT}\sum_{x=0}^{k-1} e^{-xrT}}$$

Next, we approximate $\sum_{x=0}^{k-1} e^{-xrT}$ by $\left(1-e^{-rT}\right)^{-1}$, and we approximate the summation over k by an integration over k:

$$\langle n\rangle\big|_{p,rT\ll 1} \approx \left(e^{rT} - 1\right)\int_0^{\infty} \mathrm{d}k\, e^{krT} e^{\log(1-p)e^{krT}\left(1-e^{-rT}\right)^{-1}}$$

Performing the integration and simplifying, this becomes

$$\langle n\rangle\big|_{p,rT\ll 1} \approx \frac{\left(e^{rT} - 1\right)\left(1 - e^{-rT}\right)}{-rT\log(1-p)} \tag{S9}$$

2.5.3 $0 < p \leq 1$

In the limit that p approaches 1, Equation (S9) approaches 0. In the limit that p approaches 0, Equation (S9) becomes arbitrarily large. Therefore, we can add Equations (S6) and (S9) together to obtain an approximation for $\langle n \rangle$ for any value of p:

$$\langle n \rangle \approx \frac{e^{rT} - 1}{rT} \left(1 - \frac{1 - e^{-rT}}{\log(1-p)} \right) \tag{S10}$$

3 Expected total cost per unit time

For optimizing the testing frequency, the quantity of interest is the expected total cost per unit time. The surveillance cost per unit time, C_1, is given by Equation (S1). Let C_2 denote an approximation for the expected infection cost per unit time, and let C denote an approximation for the expected total cost per unit time. We have

$$C = C_1 + C_2 \tag{S11}$$

For determining C_2, we assume that each infection contributes a cost c_2. If new lineages appear at rate λ, then C_2 is given by

$$C_2 = \lambda c_2 \langle n \rangle \tag{S12}$$

Substituting Equations (S1), (S12), and (S10) into Equation (S11), we obtain

$$C = \frac{c_1}{T} + \lambda c_2 \left(\frac{e^{rT} - 1}{rT} \right) \left(1 - \frac{1 - e^{-rT}}{\log(1-p)} \right) \tag{S13}$$

3.1 Optimal testing frequency

Equation (S13) specifies the expected total surveillance and pathogen cost per unit time. C is a function of the testing period, T, and we seek the value of T for which C is minimal. The first step is to show that C has a single minimum at a particular value of T. To do this, we differentiate C twice with respect to T:

$$\begin{aligned} \left(\frac{rT^2}{\lambda c_2} \right) \frac{\mathrm{d}C}{\mathrm{d}T} = &-\frac{rc_1}{\lambda c_2} + \left[(rT-1)e^{rT} + 1 \right] \left(1 - \frac{2}{\log(1-p)} \right) \\ &- \frac{2\left[\sinh(rT) - rT\cosh(rT) \right]}{\log(1-p)} \end{aligned} \tag{S14}$$

$$\begin{aligned} \left(\frac{rT^3}{\lambda c_2} \right) \frac{\mathrm{d}^2C}{\mathrm{d}T^2} = &\frac{2rc_1}{\lambda c_2} + \left\{ [(rT-1)^2 + 1]e^{rT} - 2 \right\} \\ &- \frac{[(rT-1)^2 + 1]e^{rT} - 2}{\log(1-p)} \\ &- \frac{[(rT+1)^2 + 1]e^{-rT} - 2}{\log(1-p)} \end{aligned} \tag{S15}$$

In Equation (S15), the quantity $rT^3/(\lambda c_2)$ is necessarily positive. If the right-hand side of Equation (S15) is positive for positive values of T, then $\mathrm{d}^2C/\mathrm{d}T^2$ is necessarily positive. Note that

$$\lim_{T\to 0}\left[\left(\frac{rT^3}{\lambda c_2}\right)\frac{\mathrm{d}^2C}{\mathrm{d}T^2}\right] = \frac{2rc_1}{\lambda c_2} > 0 \tag{S16}$$

We also have

$$\frac{\mathrm{d}}{\mathrm{d}T}\left[\left(\frac{rT^3}{\lambda c_2}\right)\frac{\mathrm{d}^2C}{\mathrm{d}T^2}\right] = r^3T^2\left[\cosh(rT) + \left(1 - \frac{2}{\log(1-p)}\right)\sinh(rT)\right] > 0 \tag{S17}$$

From Equations (S16) and (S17), it follows that the right-hand side of Equation (S15) is necessarily positive. Therefore,

$$\frac{\mathrm{d}^2C}{\mathrm{d}T^2} > 0 \tag{S18}$$

Next, note that

$$\lim_{T\to 0}\frac{\mathrm{d}C}{\mathrm{d}T} = -\infty \tag{S19}$$

We also have

$$\lim_{T\to \infty}\frac{\mathrm{d}C}{\mathrm{d}T} = \infty \tag{S20}$$

From Equations (S19), (S20), and (S18), it follows that there is a single value of T for which C is minimized.

To determine the optimal testing period, we set $\mathrm{d}C/\mathrm{d}T = 0$ and $T = T^*$ in Equation (S14). We arrive at an implicit solution for the optimal testing period, T^*:

$$\frac{rc_1}{\lambda c_2} = \left[(rT^* - 1)e^{rT^*} + 1\right]\left(1 - \frac{2}{\log(1-p)}\right) - \frac{2\left[\sinh(rT^*) - rT^*\cosh(rT^*)\right]}{\log(1-p)} \tag{S21}$$

The optimal testing frequency is given by

$$f^* = \frac{1}{T^*} \tag{S22}$$

3.1.1 Asymptotic behavior as $p \to 1$

Taking the limit $p \to 1$ in Equation (S21), we obtain the following equation for T^*:

$$\frac{rc_1}{\lambda c_2} = (rT^* - 1)e^{rT^*} + 1 \qquad (p = 1)$$

Letting $W_0(x)$ denote the principal branch of the Lambert W function, and using Equation (S22), we obtain an explicit solution for the optimal testing frequency:

$$f^* = r\left\{1 + W_0\left(\frac{1}{e}\left[\frac{rc_1}{\lambda c_2} - 1\right]\right)\right\}^{-1} \qquad (p = 1) \tag{S23}$$

Equation (S23) specifies the optimal testing frequency if $p = 1$.

3.1.2 Asymptotic behavior as $p \to 0$

For small values of p, the optimal testing frequency, T^*, is also small. To determine T^*, we consider that $p \ll 1$ and $rT^* \ll 1$ in Equation (S21). We use the approximations $\log(1-p) \approx -p$, $e^{rT^*} \approx 1 + rT^* + (rT^*)^2/2$, $\sinh(rT^*) \approx rT^* + (rT^*)^3/3!$, and $\cosh(rT^*) \approx 1 + (rT^*)^2/2$:

$$\frac{rc_1}{\lambda c_2} \approx \left[(rT^* - 1)\left(1 + rT^* + \frac{(rT^*)^2}{2}\right) + 1\right]\left(1 + \frac{2}{p}\right) + \frac{2}{p}\left[\left(rT^* + \frac{(rT^*)^3}{3!}\right) - rT^*\left(1 + \frac{(rT^*)^2}{2}\right)\right] \qquad (p \to 0)$$

Simplifying, we have

$$\frac{rc_1}{\lambda c_2} \approx \frac{(rT^*)^2}{p}\left[(1 + rT^*)\left(1 + \frac{p}{2}\right) - \frac{2rT^*}{3}\right] \qquad (p \to 0)$$

Considering that $p \ll 1$ and $rT^* \ll 1$, and using Equation (S22), we solve approximately for the optimal testing frequency:

$$f^* \sim \sqrt{\frac{r\lambda c_2}{pc_1}} \qquad (p \to 0) \tag{S24}$$

For small values of p, f^* is approximately given by Equation (S24).

4 Distribution of pathogen-related parameters

The calculation of the expected infection cost per unit time, C_2, assumes that, for each lineage that appears, the pathogen-specific parameters c_2, r, and p are the same. The expected infection cost per unit time is then just the expected cost due to a single lineage multiplied by the rate, λ, at which those lineages arise.

More generally, we can consider $\mathrm{d}c_2\,\mathrm{d}r\,\mathrm{d}p\,\lambda'(c_2, r, p)$ to be the (infinitesimal) rate at which lineages with pathogen-specific parameters c_2, r, and p appear. In this generalized model, let C_2' denote an approximation for the expected infection cost per unit time, and let C' denote an approximation for the expected total cost per unit time. We have

$$C' = C_1 + C_2' \tag{S25}$$

With knowledge of the rate density function, $\lambda'(c_2, r, p)$, we are able to compute the expected infection cost per unit time by integrating over all possible values of c_2, r, and p:

$$C_2' = \int_0^\infty \mathrm{d}c_2 \int_0^\infty \mathrm{d}r \int_0^1 \mathrm{d}p \left\{\lambda'(c_2, r, p)\, c_2 \langle n \rangle\right\} \tag{S26}$$

Substituting Equations (S1) and (S26) into Equation (S25), we obtain

$$C' = \frac{c_1}{T} + \int_0^\infty \mathrm{d}c_2 \int_0^\infty \mathrm{d}r \int_0^1 \mathrm{d}p \left\{\lambda'(c_2, r, p)\left[c_2\left(\frac{e^{rT} - 1}{rT}\right)\left(1 - \frac{1 - e^{-rT}}{\log(1-p)}\right)\right]\right\} \tag{S27}$$

The optimal testing frequency is given by

$$F^* = \frac{1}{\arg\min_T C'} \tag{S28}$$

Equations (S27) and (S28) can be solved numerically to determine the optimal testing frequency. Below, we consider several simple examples for which Equation (S27) can be solved analytically to show how the model works.

4.1 Example 1

As the simplest example of using Equation (S27), consider that only a single type of pathogen can emerge. The pathogen has per-case cost c_2', growth rate r', and probability of detection p', and new lineages are introduced at rate λ. The rate density function, $\lambda'(c_2, r, p)$, is given by

$$\lambda'(c_2, r, p) = \lambda\delta(c_2 - c_2')\delta(r - r')\delta(p - p')$$

Here, δ denotes the Dirac delta function. When this form for $\lambda'(c_2, r, p)$ is substituted into Equation (S27) and the integrations over c_2, r, and p are performed, we obtain

$$C' = \frac{c_1}{T} + \lambda c_2' \left(\frac{e^{r'T} - 1}{r'T}\right)\left(1 - \frac{1 - e^{-r'T}}{\log(1 - p')}\right)$$

Thus, Equation (S27) reduces to Equation (S13) for the case where only a single type of pathogen with fixed parameters can emerge.

4.2 Example 2

Next, consider the possibility that two different types of pathogens can emerge. Pathogen 1 has parameters c_2', r', and p', while Pathogen 2 has parameters c_2'', r'', and p''. Lineages of Pathogen 1 are introduced at rate λ_1, and lineages of Pathogen 2 are introduced at rate λ_2. The corresponding rate density function is

$$\lambda'(c_2, r, p) = \lambda_1\delta(c_2 - c_2')\delta(r - r')\delta(p - p') + \lambda_2\delta(c_2 - c_2'')\delta(r - r'')\delta(p - p'')$$

When this form for $\lambda'(c_2, r, p)$ is substituted into Equation (S27) and the integrations are performed, we obtain

$$C' = \frac{c_1}{T} + \lambda_1 c_2' \left(\frac{e^{r'T} - 1}{r'T}\right)\left(1 - \frac{1 - e^{-r'T}}{\log(1 - p')}\right) + \lambda_2 c_2'' \left(\frac{e^{r''T} - 1}{r''T}\right)\left(1 - \frac{1 - e^{-r''T}}{\log(1 - p'')}\right)$$

The expected total cost per unit time, C', is therefore equal to the surveillance cost per unit time, plus the expected infection cost per unit time for Pathogen 1, plus the expected infection cost per unit time for Pathogen 2.

4.3 Example 3

These considerations can be extended to the case where many different types of pathogens can emerge. Let Pathogen n have per-case cost $c_{2,n}$, growth rate r_n, and probability of detection p_n. The rate density function, $\lambda'(c_2, r, p)$, is given by

$$\lambda'(c_2, r, p) = \sum_n \lambda_n \delta(c_2 - c_{2,n})\delta(r - r_n)\delta(p - p_n)$$

Substituting this into Equation (S27) and integrating yields

$$C' = \frac{c_1}{T} + \sum_n \lambda_n c_{2,n} \left(\frac{e^{r_n T} - 1}{r_n T}\right)\left(1 - \frac{1 - e^{-r_n T}}{\log(1 - p_n)}\right)$$

The expected infection cost per unit time is therefore linear—i.e., we add together the expected infection costs per unit time for each of the n possible types of pathogens, and this sum equals the total expected infection cost per unit time.

4.4 Example 4

The possible parameter values that any new pathogen can have are not discrete. They are continuous. To show how this works, consider the following form for the rate density function:

$$\lambda'(c_2, r, p) = \lambda \left[\left(2\sqrt{\frac{a}{\pi}}\right) e^{-ac_2^2}\right] \delta(r - r')\delta(p - p')$$

For this case, new pathogens have growth rate r' and probability of detection p'. New pathogens can, however, have any real value of c_2 that is nonnegative. For any lineage that is introduced, its value of c_2 is most likely to be close to zero, while larger values of c_2 occur more rarely. The parameter a controls the width of the probability density function for c_2. For smaller values of a, this distribution has a longer tail, and the expected value of c_2 for any new pathogen increases. Substituting this form for the rate density function into Equation (S27) and integrating, we have

$$C' = \frac{c_1}{T} + \frac{\lambda}{\sqrt{\pi a}} \left(\frac{e^{r'T} - 1}{r'T}\right)\left(1 - \frac{1 - e^{-r'T}}{\log(1 - p')}\right)$$

4.5 Example 5

For this example, we suppose that any new pathogen has per-case infection cost c_2' and probability of detection p', while the growth rate, r, can be any nonnegative real number. We use the following form for the rate density function:

$$\lambda'(c_2, r, p) = \lambda\delta(c_2 - c_2') \left[2are^{-ar^2}\right] \delta(p - p')$$

Substituting this into Equation (S27) and integrating, we have

$$C' = \frac{c_1}{T} + \frac{\lambda c_2' \sqrt{\pi a}}{T} \left[\mathrm{erf}\left(\frac{T}{2\sqrt{a}}\right) + \left(1 - \frac{2}{\log(1 - p')}\right)\left(1 - e^{\frac{-T^2}{4a}}\right)\right] e^{\frac{T^2}{4a}}$$

4.6 Example 6

We can also model the case where new pathogens have per-case cost c_2' and growth rate r', while the probability of detection, p, can be any real number between 0 and 1. Suppose that the rate density function has the following form:

$$\lambda'(c_2, r, p) = \lambda\delta(c_2 - c_2')\delta(r - r')\left[\frac{[\theta(p-a)-1]\log(1-p)}{(1-a)\log(1-a)+a}\right]$$

Here, θ denotes the Heaviside step function. Substituting this into Equation (S27) and integrating, we obtain

$$C' = \frac{c_1}{T} + \lambda c_2'\left(\frac{e^{r'T}-1}{r'T}\right)\left(1 + \frac{a(1-e^{-r'T})}{(1-a)\log(1-a)+a}\right)$$

5 Approximation for F^*

If we have excellent understanding of the stochastic dynamics and their associated parameters, then Equations (S27) and (S28) specify the sampling frequency for which the expected total cost per unit time is minimal. However, in real settings, understanding of the underlying dynamics and parameters would only be approximate. A useful result, then, is a simple equation that approximately specifies the optimal sampling frequency and can be easily solved. Accordingly, we approximate Equation (S27) for $rT \ll 1$ as follows:

$$C' \approx \frac{c_1}{T} + \int_0^\infty \mathrm{d}c_2 \int_0^\infty \mathrm{d}r \int_0^1 \mathrm{d}p \left\{\lambda'(c_2, r, p)\left[c_2\left(1 + \frac{rT}{2}\right)\left(1 - \frac{rT}{\log(1-p)}\right)\right]\right\}$$

For small values of p, this can be approximated further:

$$C' \approx \frac{c_1}{T} + \int_0^\infty \mathrm{d}c_2 \int_0^\infty \mathrm{d}r \int_0^1 \mathrm{d}p \left\{\lambda'(c_2, r, p)\left[c_2\left(1 + \frac{rT}{p}\right)\right]\right\}$$

Differentiating with respect to T, we have

$$\frac{\mathrm{d}C'}{\mathrm{d}T} \approx \frac{-c_1}{T^2} + \int_0^\infty \mathrm{d}c_2 \int_0^\infty \mathrm{d}r \int_0^1 \mathrm{d}p \left[\lambda'(c_2, r, p)\left(\frac{c_2 r}{p}\right)\right]$$

Setting $\mathrm{d}C'/\mathrm{d}T = 0$ yields an approximation for the optimal sampling frequency:

$$F^* \approx \sqrt{\int_0^\infty \mathrm{d}c_2 \int_0^\infty \mathrm{d}r \int_0^1 \mathrm{d}p \left(\frac{c_2 r}{c_1 p}\right)\lambda'(c_2, r, p)}$$

6 Extensions of the model

In this section, we explore some simple extensions of the model.

6.1 Example 1

A realistic possibility is that the cost due to each test, c_1, is not constant but is dependent on the sensitivity of the test, p. Our model readily incorporates this generalization if we set $c_1 \to c_1(p)$ in our equations. If c_1 is an increasing function of p, then the inverse relationship between the optimal sampling frequency and p will be stronger than for the case where c_1 is constant.

6.2 Example 2

Our analytical calculation of the expected total cost per unit time is based on the assumption that the cost of an outbreak scales linearly with the number of infections. During the early stages of an outbreak, we believe this assumption to be reasonable. However, our model works the same for alternative assumptions about how the cost of an outbreak depends on the number of infections. To demonstrate this, we consider here a different possibility: that the cost of an outbreak scales quadratically with the number of infections. If we denote an approximation for the expected total cost per unit time for this scenario by C_{sq}, then this quantity is equal to

$$C_{\text{sq}} = \frac{c_1}{T} + \lambda c_2 \langle n^2 \rangle \tag{S29}$$

The task is to calculate the expected squared size of an outbreak, $\langle n^2 \rangle$. The steps in the calculation are identical to the case of linear costs.

To calculate a solution for $\langle n^2 \rangle$ for $p = 1$, we use Equation (S2), and we integrate over all possible values of τ between 0 and T:

$$\begin{aligned}
\langle n^2 \rangle\big|_{p=1} &= \int_0^T \frac{\mathrm{d}\tau}{T} \sum_{n=1}^{\infty} n^2 x_{1,n}(r\tau) \\
&= \int_0^T \frac{\mathrm{d}\tau}{T} \sum_{n=0}^{\infty} n^2 \left(1 - e^{-r\tau}\right)^{n-1} e^{-r\tau} \\
&= \frac{1}{rT} \int_0^T \mathrm{d}\tau \frac{\mathrm{d}}{\mathrm{d}\tau} \sum_{n=0}^{\infty} n(1 - e^{-r\tau})^n \\
&= \frac{1}{rT} \int_0^T \mathrm{d}\tau \frac{\mathrm{d}}{\mathrm{d}\tau} \left[(1 - e^{-r\tau}) \sum_{n=0}^{\infty} n(1 - e^{-r\tau})^{n-1} \right] \\
&= \frac{1}{rT} \int_0^T \mathrm{d}\tau \frac{\mathrm{d}}{\mathrm{d}\tau} \left[(e^{r\tau} - 1) \left(\frac{1}{r}\right) \frac{\mathrm{d}}{\mathrm{d}\tau} \sum_{n=0}^{\infty} (1 - e^{-r\tau})^n \right] \\
&= \frac{1}{rT} \int_0^T \mathrm{d}\tau \frac{\mathrm{d}}{\mathrm{d}\tau} \left[(e^{r\tau} - 1) \left(\frac{1}{r}\right) \frac{\mathrm{d}}{\mathrm{d}\tau} (e^{r\tau}) \right] \\
&= \frac{1}{rT} \int_0^T \mathrm{d}\tau \frac{\mathrm{d}}{\mathrm{d}\tau} \left[e^{r\tau} \left(e^{r\tau} - 1\right) \right]
\end{aligned}$$

Performing the integration, we obtain

$$\langle n^2 \rangle\big|_{p=1} = \frac{e^{rT} \left(e^{rT} - 1\right)}{rT} \tag{S30}$$

For $p \ll 1$ and $rT \ll 1$, we have the following result for the expected size of an outbreak:

$$\langle n^2 \rangle\big|_{p,rT\ll 1} \approx q^{e^{rT}}\left(e^{4rT} - e^{2rT}\right) + q^{e^{rT}} q^{e^{2rT}}\left(e^{6rT} - e^{4rT}\right) + q^{e^{rT}} q^{e^{2rT}} q^{e^{3rT}}\left(e^{8rT} - e^{6rT}\right) + \cdots$$

More compactly:

$$\langle n^2 \rangle\big|_{p,rT\ll 1} \approx \left(e^{2rT} - 1\right) \sum_{k=1}^{\infty} e^{2krT} q^{e^{krT} \sum_{x=0}^{k-1} e^{-xrT}}$$

Next, we approximate $\sum_{x=0}^{k-1} e^{-xrT}$ by $\left(1 - e^{-rT}\right)^{-1}$, and we approximate the summation over k by an integration over k:

$$\langle n^2 \rangle\big|_{p,rT\ll 1} \approx \left(e^{2rT} - 1\right) \int_0^{\infty} \mathrm{d}k\, e^{2krT} e^{\log(1-p) e^{krT}\left(1-e^{-rT}\right)^{-1}}$$

This can be rewritten as

$$\langle n^2 \rangle\big|_{p,rT\ll 1} \approx \frac{\left(e^{2rT} - 1\right)\left(1 - e^{-rT}\right)}{rT \log(1-p)} \int_0^{\infty} \mathrm{d}k\, e^{krT} \frac{\mathrm{d}}{\mathrm{d}k}\left(e^{\log(1-p) e^{krT}\left(1-e^{-rT}\right)^{-1}}\right)$$

Integrating by parts, we have

$$\langle n^2 \rangle\big|_{p,rT\ll 1} \approx \frac{\left(e^{2rT} - 1\right)\left(1 - e^{-rT}\right)}{-\log(1-p)} \int_0^{\infty} \mathrm{d}k\, e^{krT} e^{\log(1-p) e^{krT}\left(1-e^{-rT}\right)^{-1}}$$

Performing the integration and simplifying, this becomes

$$\langle n^2 \rangle\big|_{p,rT\ll 1} \approx \frac{e^{2rT} - 1}{rT}\left(\frac{1 - e^{-rT}}{\log(1-p)}\right)^2 \tag{S31}$$

In the limit that p approaches 1, the above expression approaches 0. In the limit that p approaches 0, the above expression becomes arbitrarily large. Therefore, we can add Equations (S30) and (S31) together to obtain an approximation for $\langle n^2 \rangle$ for any value of p:

$$\langle n^2 \rangle \approx \frac{e^{rT}\left(e^{rT} - 1\right)}{rT} + \frac{e^{2rT} - 1}{rT}\left(\frac{1 - e^{-rT}}{\log(1-p)}\right)^2 \tag{S32}$$

Substituting Equation (S32) into Equation (S29), we obtain

$$C_{\mathrm{sq}} = \frac{c_1}{T} + \lambda c_2 \left[\frac{e^{rT}\left(e^{rT} - 1\right)}{rT} + \frac{e^{2rT} - 1}{rT}\left(\frac{1 - e^{-rT}}{\log(1-p)}\right)^2\right] \tag{S33}$$

Equation (S33) specifies the expected total cost per unit time for the case where the cost due to a single outbreak scales quadratically with the number of infections.

6.3 Example 3

Our model can be adapted to the case where breakthrough infections are a possibility. As a simple example, we suppose that immediately after an outbreak is detected and intervention is applied, a single new infection is initiated with probability b, while with probability $1-b$, there are no follow-up infections. The approximate expected total cost per unit time in this scenario, which we denote C_b, is given by

$$C_b = \frac{c_1}{T} + \lambda c_2 \left[\langle n \rangle + (1-b) \sum_{k=0}^{\infty} b^k k \langle n \rangle_{\tau=T} \right] \tag{S34}$$

$\langle n \rangle_{\tau=T}$ denotes the expected size of an outbreak, given that the first environmental test is performed at time T after the outbreak begins. The summation in square brackets is understood as follows:

- With probability $1-b$, all infections of the original outbreak are controlled, and there are no further pathogen-related costs. The expected total cost related to the original outbreak is equal to $c_2 \langle n \rangle$.
- With probability $b(1-b)$, a single case of the original outbreak is not controlled. It then leads to a second outbreak, and the pathogen must again be detected and controlled. The expected size of the second outbreak when it is detected is equal to $\langle n \rangle_{\tau=T}$. The expected total cost related to the original outbreak and the second outbreak is equal to $c_2(\langle n \rangle + \langle n \rangle_{\tau=T})$.
- With probability $b^2(1-b)$, a single case of the original outbreak is not controlled. It then leads to a second outbreak, and the pathogen must again be detected and controlled. The expected size of the second outbreak when it is detected is equal to $\langle n \rangle_{\tau=T}$ However, a single case of the second outbreak is not controlled, leading to a third outbreak. The expected size of the third outbreak when it is detected is equal to $\langle n \rangle_{\tau=T}$. The expected total cost related to the original outbreak, the second outbreak, and the third outbreak is equal to $c_2(\langle n \rangle + 2\langle n \rangle_{\tau=T})$.

The understanding is similar for higher-order terms in the summation in Equation (S34). This equation can be rewritten:

$$C_b = \frac{c_1}{T} + \lambda c_2 \left[\langle n \rangle + \langle n \rangle_{\tau=T}\, b(1-b) \sum_{k=0}^{\infty} k b^{k-1} \right]$$

This is equal to

$$C_b = \frac{c_1}{T} + \lambda c_2 \left[\langle n \rangle + \langle n \rangle_{\tau=T}\, b(1-b) \frac{\mathrm{d}}{\mathrm{d}b} \sum_{k=0}^{\infty} b^k \right]$$

This becomes

$$C_b = \frac{c_1}{T} + \lambda c_2 \left[\langle n \rangle + \langle n \rangle_{\tau=T}\, b(1-b) \frac{\mathrm{d}}{\mathrm{d}b} \left(\frac{1}{1-b} \right) \right]$$

Simplifying, we have

$$C_b = \frac{c_1}{T} + \lambda c_2 \left[\langle n \rangle + \langle n \rangle_{\tau=T} \left(\frac{b}{1-b} \right) \right] \tag{S35}$$

For $p = 1$, $\langle n \rangle_{\tau=T}$ is given by

$$\langle n \rangle_{\tau=T} \Big|_{p=1} = e^{rT} \tag{S36}$$

For $p \ll 1$ and $rT \ll 1$, $\langle n \rangle_{\tau=T}$ is given by Equation (S9):

$$\langle n \rangle_{\tau=T} \Big|_{p,rT \ll 1} \approx \frac{\left(e^{rT} - 1\right)\left(1 - e^{-rT}\right)}{-rT \log(1-p)} \tag{S37}$$

In the limit that p approaches 1, Equation (S37) approaches 0. In the limit that p approaches 0, Equation (S37) becomes arbitrarily large. Therefore, we can add Equations (S36) and (S37) together to obtain an approximation for $\langle n \rangle_{\tau=T}$ for any value of p:

$$\langle n \rangle_{\tau=T} \approx e^{rT} - \frac{\left(e^{rT} - 1\right)\left(1 - e^{-rT}\right)}{rT \log(1-p)} \tag{S38}$$

Substituting Equations (S10) and (S38) into Equation (S35), we obtain

$$\begin{aligned} C_b = {} & \frac{c_1}{T} \\ & + \lambda c_2 \left[\left(\frac{e^{rT} - 1}{rT} \right) \left(1 - \frac{1 - e^{-rT}}{\log(1-p)} \right) + \left(e^{rT} - \frac{\left(e^{rT} - 1\right)\left(1 - e^{-rT}\right)}{rT \log(1-p)} \right) \left(\frac{b}{1-b} \right) \right] \end{aligned} \tag{S39}$$

Equation (S39) specifies the expected total cost per unit time for the case where, whenever an outbreak is detected, a single new infection breaks through control and is initiated with probability b, while intervention eliminates all infections with probability $1 - b$.
